# Engineering Efficient Self-Assembled Plasmonic Nanostructures by Configuring Metallic Nanoparticle’s Morphology

**DOI:** 10.3390/ijms221910595

**Published:** 2021-09-30

**Authors:** Vasanthan Devaraj, Jong-Min Lee, Ye-Ji Kim, Hyuk Jeong, Jin-Woo Oh

**Affiliations:** 1 Bio-IT Fusion Technology Research Institute, Pusan National University, Busan 46241, Korea; devarajvasanthan@gmail.com (V.D.); jongminlee1984@gmail.com (J.-M.L.); marionsal0926@gmail.com (H.J.); 2 School of Nanoconvergence Technology, Hallym University, Chuncheon 24252, Korea; 3 Department of Nano Fusion Technology and BK21 Plus Nano Convergence Division, Pusan National University, Busan 46241, Korea; kkyeaj0608@gmail.com; 4 Department of Nanoenergy Engineering, Pusan National University, Busan 46241, Korea

**Keywords:** self-assembly, metallic nanoparticles, plasmonic modes, simulations, surface charge mappings, full-width three-quarter maximum

## Abstract

We reveal the significance of plasmonic nanoparticle’s (NP) shape and its surface morphology en route to an efficient self-assembled plasmonic nanoparticle cluster. A simplified model is simulated in the form of free-space dimer and trimer nanostructures (NPs in the shape of a sphere, cube, and disk). A ~200% to ~125% rise in near-field strength (gap mode enhancement) is observed for spherical NPs in comparison with cubical NPs (from 2 nm to 8 nm gap sizes). Full-width three-quarter maximum reveals better broad-spectral optical performance in a range of ~100 nm (dimer) and ~170 nm (trimer) from spherical NPs as compared to a cube (~60 nm for dimer and trimer). These excellent properties for sphere-based nanostructures are merited from its dipole mode characteristics.

## 1. Introduction

Plasmonic nanoparticles (NPs) have received various attention as they introduce interesting optical properties at sub-wavelength scale [1,2,3,4,5]. NPs, either by ordered or self-assembled distribution, can manipulate light–matter interactions and generate enhanced near-field properties leading to various applications in the field of surface-enhanced Raman spectroscopy (SERS), non-linear optics, sensors, non-classical light sources, energy, artificial magnetism, and so on [6,7,8,9,10,11,12]. The local field or near-field enhancement opens up an attractive optical property strongly relying upon the optical resonance of metallic nanostructures. These significantly enhance the electromagnetic field, mainly due to surface plasmon resonance (SPR) [13,14,15,16,17]. The electromagnetic field or near-field enhancement in plasmonic materials has generated significant interest in understanding various plasmonic modes [18,19,20,21].

Even though recent advances in top-down fabrication approaches can lead to highly efficient plasmonic devices, it involves multiple/complex processing steps alongside cost burdens [22,23,24]. In addition to the above, uniformity in achieving mass production or distribution of plasmonic nanostructures with identical gap sizes will be difficult. However, on the other side, the self-assembly approach provided an opportunity to build these nanostructures in a versatile, low-cost path [25,26,27,28,29,30]. Self-assembled plasmonic NP clusters and SERS substrates are few such examples developed through this strategy. Factors such as geometrical shape, size, material choice, doping, and surroundings (ex., such as a coated surface layer with different refractive index material) play a vital role in manipulating plasmonic properties in self-assembled nanostructures based upon application requirements [1,4,5,6,7,13,16,17,18,19,20]. It is essential to consider that this generated near-field is not uniformly distributed all over nanostructures but relatively highly localized in spatially narrow regions such as interparticle nanogaps, nanotips, or NP-spacer nanogaps, which were called hot-spots [1,5,6,7,16,17,18,19,20,31,32]. One of the critical properties in effectively optimizing the hot-spot region will be the NP shape or surface morphology. In optimizing hot-spot, NP shapes such as sphere, disk, and cube play an essential role, as reported in the vast literature [5,6,13,14,15,16,17,18,25,26,27,28,33,34,35,36,37]. In particular, NP shapes such as spheres and cubes can help to fabricate self-assembled plasmonic nanostructures, owing to their commercial availability. In developing an efficient design guideline, simulating large area self-assembled nanostructures (considering nanoscale meshing size for accurate results) will be a hectic and complex task, as it will take an enormous amount of time alongside costlier super-sized server build(s). We can consider simplified models that can quickly bring precise solutions without relying on super-computers to solve this issue. 

In this work, we numerically investigated the sphere-, disk-, and cube-based NP’s plasmonic properties ranging from dimer to trimer and discussed its results. We considered disk NP (example from top-down approach) as a reference while exploring and evaluating sphere- and cube-based NP’s (easy to utilize in forming self-assembled clusters) plasmonic properties. These dimer and trimer nanostructures can act as a simplified model of self-assembled NP clusters or SERS substrates in understanding the design optimization process of an efficient plasmonic nanostructure. Our simulation studies reveal that sphere-based nanostructures can be advantageous considering a self-assembly-based approach. These results will open insights into the proper utilization of NP shapes and their incorporation towards highly efficient plasmonic nanostructures.

## 2. Modeling Information 

### 2.1. Near-Field Calculation 

**Figure 1:**
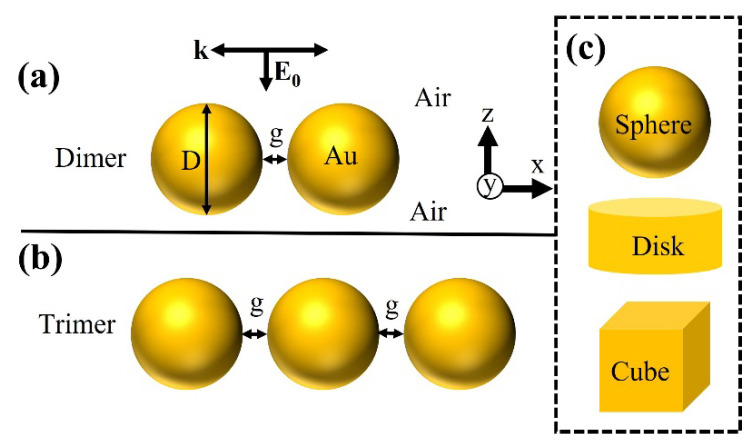
Schematic illustration of free-space (**a**) dimer and (**b**) trimer NPs separated by gap size “g” and diameter “D”. Plane-wave source illuminate plasmonic nanostructures in the normal direction with an incident electric field of E_0_. (**c**) NP shapes modeled in this work are sphere, disk, and cube. Detailed information on modeling conditions is described in Supplementary Materials or SM Figure S1.

For near-field calculations, a maxwell electromagnetic solver from ANSYS Lumerical FDTD solutions was employed. This work considered free-space Au nanoparticles (NPs) with a fixed diameter “D” of 100 nm. Dimer and trimer NPs in the shape of a sphere, disk, and cube are modeled, as they were extensively studied for various plasmonic applications (Figure 1, Supplementary Materials or SM Figure S1). Interparticle distance or gap size “g” is varied from 2 nm to 50 nm. A broadband plane-wave source from normal direction (+Z) excites free-space metallic NPs to study the optical properties from the nanostructure. A meshing size of 0.3 nm is employed to extract highly accurate results. A perfectly matched layers (PML) boundary condition is applied in the XYZ direction. Au’s refractive index is extracted from Johnson and Christy database [38]. A box-shaped power monitor is placed close to the nanostructures to record the near-field properties. For the calculation of near-field properties, we considered an average volume integral of |E/E_0_| [39,40,41,42,43]:

(1) $$\mathrm{Near}-\mathrm{field}\;\mathrm{enhancement}=\frac{∭\;\left|E/E_{0}\right|\mathrm{dV}}{V}\;$$

From Equation (1), the amplitude of incident electric field is given by E_0_ (modulus of incident field |E_0_| = 1 V/m), generated local electric field is E = (E_x_, E_y_, E_z_) and volume at a certain distance above the metallic NP surface is given by V. 

### 2.2. Three-Dimensional Surface Charge Mappings

Complicated optical modes in plasmonic nanostructures can be effectively understood when extracting three-dimensional surface charge mappings (3DSCM). Taking the skin effect into consideration and applying an integration of Gauss’s law, it is possible to calculate the surface charge density (ρ). Considering skin depth δ, an induced charge density (ρ_r_) at the surface (S) of the metal (r is the depth from the surface) and total polarization charge Q = 0 within NP, the following equation can be derived:

(2) $$\begin{array}{c} Q=∭\rho_{r}drdS=∭\rho e^{-r/\delta }\;drdS\;\\ =∯\rho dS\;\int_{0}^{R}e^{-r/\delta }dS\\ =\delta \;\left(1-e^{-R/\delta }\right)\;{∯}_{S}\rho dS\;\; \end{array}$$

Here, radius of NP is given as “R”. Further, utilizing an integral form of Gauss’s law:

(3) ΦE=Qε0= ∯S(n· E) dS= ∯S(nx · Ex+ ny· Ey+ nz· Ez) dS

From Equation (3), Φ_E_ is the electric flux through the metal surface S, the outward normal vector is given as *n* = (n_x_, n_y_, n_z_), local electric field is E = (E_x_, E_y_, E_z_) and permittivity of vacuum ε_0_. Considering above all factors, the surface charge density can be derived as follows:

(4) $$\rho =\;\frac{\varepsilon_{0}\;\left(n_{x}\;\cdot \;E_{x}+\;n_{y}\cdot \;E_{y}+\;n_{z}\cdot \;E_{z}\right)}{\delta \left(1-\;e^{-R/\delta }\right)}\;\alpha \;\left(n_{x}\cdot \;E_{x}+\;n_{y}\cdot \;E_{y}+\;n_{z}\cdot \;E_{z}\right)$$

As seen from Equation (4), the surface charge density ρ is given as (n_x_·E_x_ + n_y_·E_y_ + n_z_·E_z_) [16,20,31,44,45]. Utilizing this surface charge mapping approach, we directly extracted the 3DSCM from our simulations using the COMSOL Multiphysics tool (Wave optics module).

## 3. Results and Discussion 

**Figure 2:**
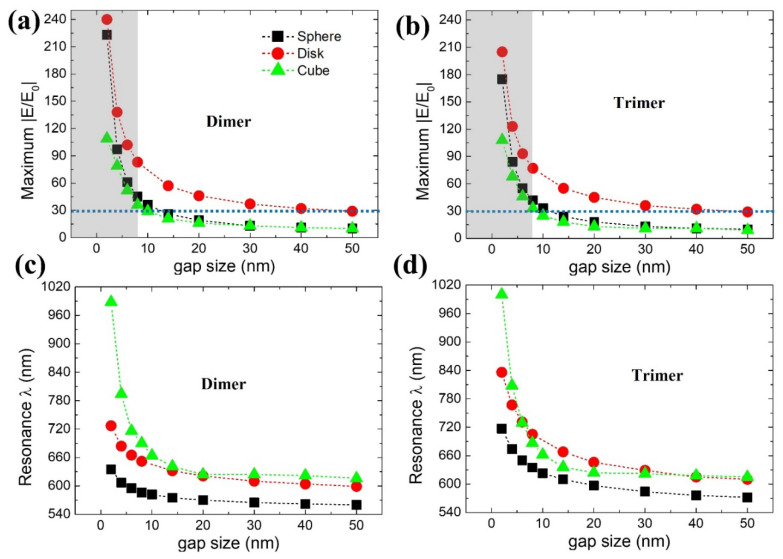
Maximum near-field strength |E/E_0_| obtained from dimer (**a**) and trimer (**b**) plasmonic nanostructures for different NP shapes (sphere, disk, and cube) from its respective resonance wavelength positions (**c**) and (**d**), respectively. These data are extracted from simulated broadband |E/E_0_| spectra as seen from SM Figures S2 and S3. Grey color region within blue dotted lines indicates our region of interest on the basis of gap mode enhancement.

Figure 2 shows modeled gap size-dependent maximum near-field strength (dimer—Figure 2a, trimer—Figure 2b) properties extracted from its resonance wavelength positions (dimer—Figure 2c, trimer—Figure 2d). For complete broadband near-field spectra related to Figure 2, please see SM Figure S2 (dimer nanostructures) and Figure S3 (trimer nanostructures). We have to consider two scenarios when interpreting dimer versus trimer plasmonic properties as a function of gap size “g”: (i) resonance wavelength shift and (ii) deterioration in near-field enhancement |E/E_0_| strength. For varied gap sizes between 2 nm to 50 nm, dimer nanostructure’s (Figure 2a) |E/E_0_| deteriorated as following: 223 to 10 (sphere), 240 to 29 (disk), and 109 to 10 (cube). In addition to the above, resonance wavelength tuning range as a function of gap size are as follows: 75 nm (635 nm to 560 nm), 128 nm (727 nm to 599 nm), 372 nm (988 nm to 616 nm) for sphere, disk, and cube nanostructures, respectively (Figure 2c). Interestingly, in trimer nanostructure(s), increases in |E/E_0_| were not seen as compared to dimer(s), especially at smaller gap sizes (for example, until g = 8 nm). Alternatively, in other words, an increase in the number of NPs does not directly relate to a rise in near-field enhancement (for trimer nanostructure, observed |E/E_0_| at 2 nm gap distance were 175 (sphere), 205 (disk), and 108 (cube), respectively). Nevertheless, at the same time, relatively more comprehensive resonance wavelength tuning range at similar gap size differences are noted for trimer: 145 nm (717 nm to 572 nm), 226 nm (836 nm to 610 nm), 385 nm (1000 nm to 615 nm) for sphere, disk, and cube nanostructures, respectively (Figure 2d). From this point, we will consider and interpret these nanostructures’ plasmonic properties until g = 8 nm condition (grey color shaded part, Figure 2a,b) considering the absence of gap mode in spherical NPs [39,40].

**Figure 3:**
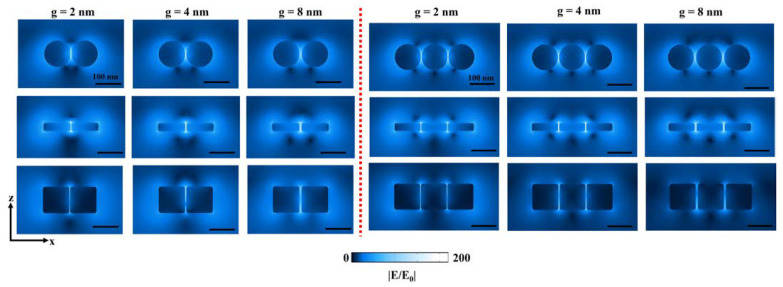
Cross-section XZ electric field profiles taken at g = 2 nm, 4 nm, and 8 nm for a dimer (**left**) and trimer (**right**) nanostructures studied in this work. Better near-field enhancement can be observed from sphere- and disk-based nanostructures when compared to a cube. Electric field profiles are scaled to the same color strength for a fair comparison. All scale bars measure 100 nm.

The differences in |E/E_0_| for dimer and trimer properties are in good agreement with the obtained cross-section electric field profiles (Figure 3). Clearly, dimer structures reveal better |E/E_0_| characteristics when compared with trimers. Therefore, our justification for identifying a good design approach for self-assembled plasmonic NP clusters with dimer and trimer nanostructures can be reasonable. In general, disk nanostructure is advantageous considering near-field enhancement deterioration rate when “g” is varied from smaller to larger size. However, on the contrary, spherical NPs seem to outperform cubical nanostructures in terms of |E/E_0_| when considering smaller gap sizes until 8 nm. It is necessary to understand the reason behind this nature, as it can play a vital role in various applications. It is not easy to interpret by only considering electric field profiles. Therefore, we considered three-dimensional surface charge mappings or 3DSCM, which will help understand any complex plasmonic properties behind these notable differences. SM Figure S4 depicts the schematic plasmonic mode charge distribution profiles close to the cavity or “g” region (not to scale) based on dipole (solid circle) and quadrupole (solid triangle) modes. These solid circle or triangle symbols apply to all our 3DSCM data in identifying plasmonic modes.

**Figure 4:**
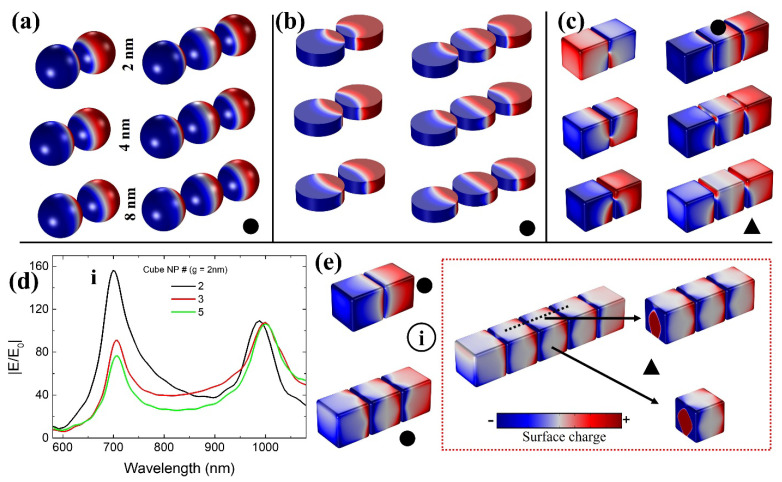
Simulated 3DSCM profiles taken from dimer and trimer nanostructures with different gap sizes for NP shapes of a sphere (**a**), disk (**b**), and cube (**c**). Solid circle (dipole) and triangle (quadrupole) symbols represent the plasmonic modes. All 3DSCM profiles are arranged in order of following gap size of 2 nm, 4 nm, and 8 nm. (**d**) Broadband |E/E_0_| spectra taken from cube nanostructures with increasing NP numbers from two to five and respective (**e**) 3DSCM profiles extracted from the position marked as “i”.

The 3DSCM properties of dimer and trimer nanostructures based on a sphere, disk, and cube are shown in Figure 4a–c for g = 2 nm, 4 nm, and 8 nm sizes. Dipole mode characteristics (solid circles) were seen from sphere and disk nanostructures. However, in the case of cubical nanostructures, inconsistent mode properties were observed. In dimer nanostructures (Figure 4c), quadrupole mode properties (solid triangle) were present despite different gap sizes. Regarding the trimer, dipole mode was observed at g = 2 nm but deteriorated to quadrupole mode with increasing gap size. Poor |E/E_0_| properties in cubical nanostructures can be understood as they lack the dipole mode characteristics. 

Furthermore, |E/E_0_| properties at shorter wavelength positions (dimer = 699 nm and trimer = 707 nm, marked as “i” in Figure 4d) were not considered a dominant or primary gap mode position for cubical NPs. When “g” is increased, resonance wavelength shift should generally follow from longer to shorter wavelength positions in terms of gap mode, especially concerning smaller “g” variations. When gap size varied from 2 nm to 8 nm, |E/E_0_| resonance wavelength shift from 988 nm (1000 nm) to 690 nm (687 nm) makes sense in dimer (trimer) nanostructures rather than the position marked as “i” in Figure 4d. Adding to the above complexity, changes from dipole to quadrupole mode were noted with an increased number of cubes until five (dipole mode disappeared when there were five NPs, Figure 4e). Therefore, what can be an issue with cube-based nanostructures in displaying comparatively lower near-field strength? How do disk and sphere NPs perform better at smaller gap sizes (2 nm to 8 nm)? The optical background behind this phenomenon should be studied. 

One possible significant interpretation can be envisioned when we see it in terms of NP facet(s). Facets in an NP will play a vital role in enhancing gap mode-based |E/E_0_| characteristics. Dipole mode properties and better |E/E_0_| strength at resonance position can be achieved with fewer facets in NPs [45]. The presence of more facets in NP deteriorates the plasmonic properties. Higher |E/E_0_| performance in disk nanostructures can be seen as they have only two facets, but in the cube case, four facets were reasonable in deterioration of its dipole mode properties alongside near-field enhancement. It is well known that gap mode is absent in sphere-based nanostructures at a larger gap size, but within smaller gap sizes (for example, g ≤ 6 nm or 8 nm), it is possible to realize better near-field properties [39,40]. Rather than having more facets, it is possible to extract higher |E/E_0_| even with smoother NP surfaces, such as a sphere. Considering these factors, we can assume the problem lies with more facets in cubical nanostructures compared with disk or sphere.

**Figure 5:**
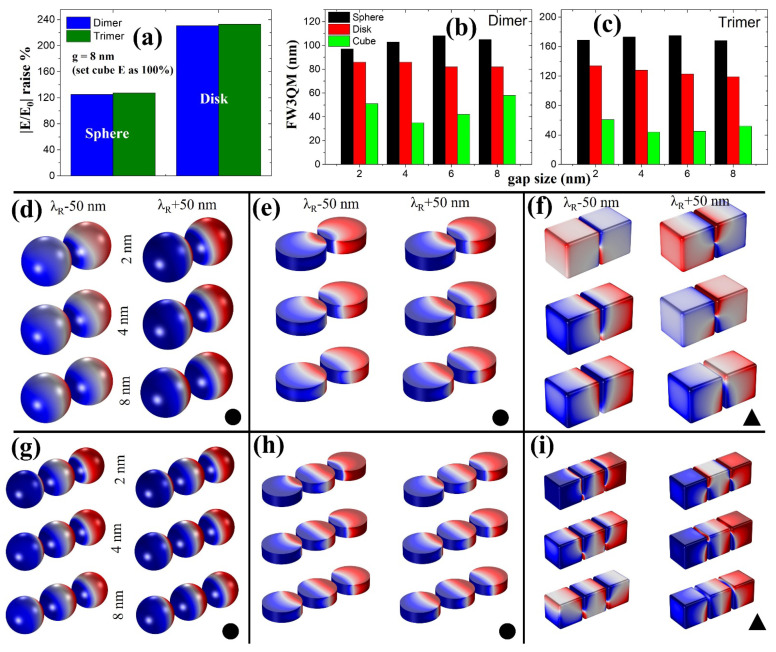
(**a**) Calculated |E/E_0_| raise% for sphere- and disk-based dimer and trimer NPs at g = 8 nm compared to a cube. (**b**) FW3QM results of dimers (**b**) and trimers (**c**) for different gap sizes ranging from 2 nm to 8 nm reveals sphere-based NPs can perform better. The 3DSCM profiles indicating plasmonic mode characteristics of a sphere, disk, and cube NPs taken at λ_R_ ± 50 nm wavelength positions for dimer (**d**–**f**) and trimer (**g**–**i**) nanostructures. Here λ_R_ represents resonance wavelength position as seen from Figure 1c,d. All 3DSCM profiles are arranged to follow gap sizes of 2 nm, 4 nm, and 8 nm. Solid circle (dipole) and triangle (quadrupole) symbols represent the plasmonic modes. In 3DSCM profiles, blue represents negative charge and red denotes positive charge.

To understand these differences and further analyze them, we considered the following two optical properties: (i) |E/E_0_| raise% and (ii) full-width three-quarter maximum or FW3QM (Figure 5a–c). In case of |E/E_0_| raise%, we fixed g = 8 nm |E/E_0_| strength of cube as 100% and calculated the how much increase or decrease in |E/E_0_| percentage could be extracted with sphere and disk nanostructures (please note that g = 8 nm fixed throughout the nanostructures for a fair comparison). Sphere- and disk-based dimer/trimer nanostructures displayed 125%/127% and 231%/233% increase in |E/E_0_| strength, respectively (Figure 5a). Please note that for reference, even at g = 2 nm, ~200% |E/E_0_| raise% is possible for sphere NPs as compared to a cube. 

For FW3QM, a 75% maximum |E/E_0_| strength obtained from the respective resonance wavelength position is considered (Figure 2a,b) for the plasmonic nanostructures (gap sizes from 2 nm to 8 nm). For detailed information, please see SM Figure S5 and SM Tables S1 and S2 explaining our calculation method. Sphere-based dimer (trimer) nanostructures showed excellent FW3QM ranging 97 nm, 103 nm, 108 nm, and 105 nm (169 nm, 173 nm, 175 nm, and 168 nm), respectively. Disk-based dimer (trimer) nanostructures showed FW3QM ranging 86 nm, 86 nm, 82 nm, and 82 nm (134 nm, 128 nm, 123 nm, and 119 nm), respectively. Poor FW3QM characteristics from cube-based dimer (trimer) nanostructures can be seen as follows: 51 nm, 35 nm, 42 nm, and 58 nm (61 nm, 44 nm, 45 nm, and 52 nm). Please note that these FW3QM are obtained from “g” sizes of 2 nm, 4 nm, 6 nm, and 8 nm, respectively, in order. A better broad-spectral range in FW3QM can be noted with an increased number of NPs for sphere and disk nanostructures compared with a cube. Please note that by achieving a broader FW3QM spectral range, deterioration in optical efficiency can be minimized (even with fewer fabrication errors from optimum design point) when considering cavity-based applications [45,46,47]. It is critical to have such a broader spectral range of FW3QM, where various highly efficient optical applications can be realized in the field of non-classical light emitters, surface-enhanced Raman spectroscopy, sensors, and quantum dot-based devices [45,46,47,48]. From 3DSCM, we can see dipole mode characteristics for sphere and disk-based nanostructures in a 100 nm wavelength span for different gap sizes (Figure 5d,e,g,h). Deterioration in cube-based nanostructure’s plasmonic properties can be seen due to the dominant presence of quadrupole mode at an identical geometrical condition (Figure 5f,i). 

**Figure 6:**
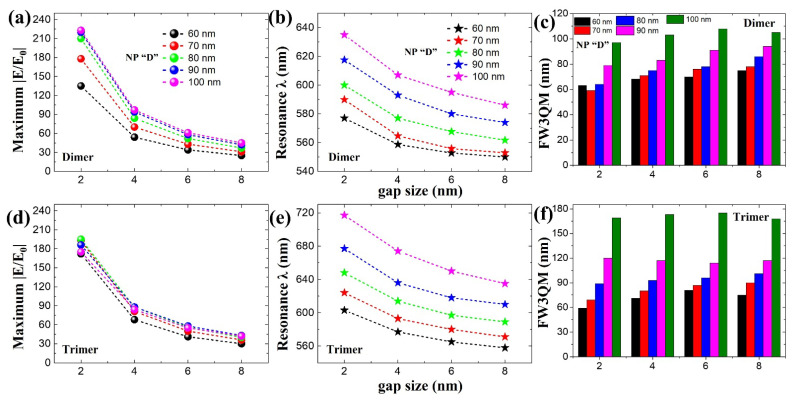
Size-dependent spherical NP’s plasmonic properties for (**a**–**c**) dimer and (**e**,**f**) trimer nanostructures. NP “D” is varied from 60 nm to 100 nm. Maximum near-field strength |E/E_0_| obtained from dimer (**a**) and trimer (**e**) plasmonic nanostructures from its respective resonance wavelength positions (**b**) and (**e**), respectively. FW3QM results of dimers (**c**) and trimers (**f**) for different gap sizes ranging from 2 nm to 8 nm as a function for different NP “D” sizes. These data are extracted from simulated broadband |E/E_0_| spectra from SM Figures S6 and S7.

We further studied size-dependent (NP diameter “D”) plasmonic properties for a sphere-based dimer (Figure 6a–c) and trimer (Figure 6d–f) nanostructures. Figure 6 reveals the plasmonic properties of sphere-based nanostructures for different NP “D” ranging from 60 nm to 100 nm. Gap sizes within a range of 2 nm to 8 nm were considered in these simulations following Figure 1, where we can still observe gap mode-based near-field enhancement. By increasing NP “D”, we can observe resonance wavelength positions moving towards longer wavelength regions: from 577 nm to 635 nm (dimer, Figure 6a) and from 603 nm to 717 nm (trimer, Figure 6d) for a gap size of 2 nm (as an example). In the case of |E/E_0_|, dimer nanostructures reveal a steady rise in near-field enhancement properties until D = 90 nm and gradually become constant after that (Figure 6b). In the case of trimer nanostructures, rather than a steady increase in |E/E_0_|, we can see constant and ~ consistent near-field enhancement characteristics (Figure 6e). Maximum broad-spectral ranges of 105 nm (dimer) and 175 nm (trimer) are possible considering FW3QM calculations (Figure 6c,f). Considering these results, we believe that dimer- or trimer-based designs can guide us to approximately design or evaluate self-assembled plasmonic nanoclusters. 

**Figure 7:**
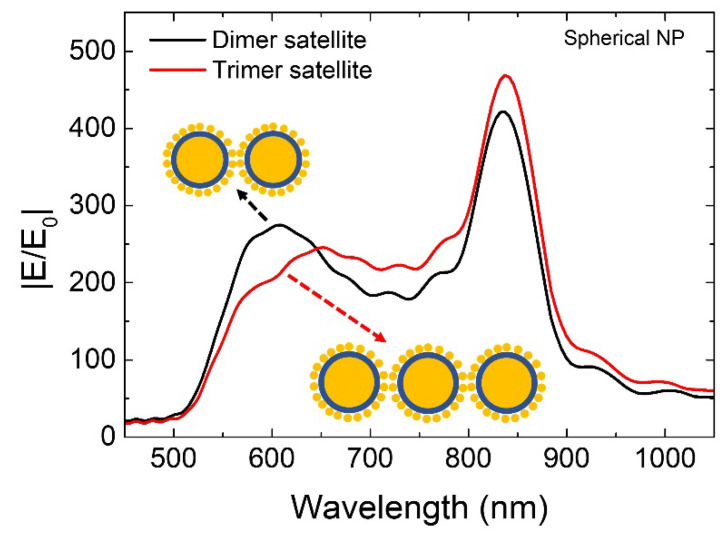
Simulated broadband near-field enhancement properties from core-shell satellite nanostructures.

We extended our simulation studies to a simple core-shell satellite structure model (Figure 7). Bigger NP with D of 100 nm surrounded by smaller NP with D of 10 nm is modeled. A thin dielectric coating (*n* = 1.45) over bigger NP with a thickness of 2 nm is considered (blue colored layer as shown in Figure 7). We observed ~two-fold times increase in near-field enhancement compared to bare dimer/trimer nanostructures, which we believe is highly beneficial for various applications. At the same time, it is also possible to study and understand various super- and sub-radiant modes from these satellite structures [1,26,27,28,29]. 

Our results show a critical understanding of metallic NP’s optical properties to determine which geometrically-shaped NPs are needed in self-assembling efficient plasmonic nanostructures. The role of NP shape and its surface morphology effectively controls the enhancement of electromagnetic fields originating from nanoscale features such as interparticle distance, edges, tips, or crevices. Even though disk-based nanostructures fared better in achieving |E/E_0_| raise percentage, we can choose spherical NPs, considering self-assembly advantages, which facilitates simple, low-cost fabrication. Full-width three-quarter maximum results prove how sphere-based nanostructures can have a broader spectral width advantage over the disk or cube-based NPs, which can be important for applications like non-classical light sources. The deteriorating gap mode strength as a function of gap size in cubical nanostructures is significantly related to the presence of more facets (than disk or sphere) alongside quadrupole mode characteristics. In other words, plasmonic nanostructures with dominant dipole mode characteristics can perform better than higher-order mode(s). With recent developments in spherical NPs, if we can precisely synthesize single faceted spherical NPs (rest surface being smoother), near-field strength can be boosted further with a slower deterioration rate extending to larger gap sizes [45]. This discussion only performed relative comparisons between building blocks (sphere and cube) of self-assembled plasmonic structures, and plasmon damping due to radiation was not considered. In addition, future studies involving plasmon damping (related to charge carriers in metals), can open up further understanding of nanoplasmonics properties [49]. We believe optical insights revealed in this work can open various applications in self-assembled plasmonic NP clusters, SERS, light-harvesting, ultrafast optoelectronic devices, and sensors.

## 4. Conclusions

In summary, our simulation studies reveal how the shape and surface morphology of plasmonic NPs can influence the near-field enhancement properties. In the presence of gap mode, spherical NPs can perform better than cubical NPs in terms of |E/E_0_| raise% (~200% and ~25% increase at g = 2 nm and 8 nm). From FW3QM, spherical NPs displayed excellent broad-spectral range near-field enhancement properties at a maximum of ~160 nm with the presence of dipole mode across this wavelength range. Poor performance in cube-based nanostructures can be related to its facet numbers and quadrupole mode characteristics. Utilizing spherical NPs in making self-assembled NP clusters or SERS substrates can be advantageous over other NP shapes such as a cube, considering the following advantages: efficient near-field generation in the presence of broad-spectral dipole mode distribution, simplicity in fabrication, and low-cost factor supporting large scale self-assembly.

## Supplementary Materials

The following are available online at https://www.mdpi.com/article/10.3390/ijms221910595/s1.
