# Supplementary material for: Engineering Efficient Self-Assembled Plasmonic Nanostructures by Configuring Metallic Nanoparticle’s Morphology
Source: Int J Mol Sci. 2021 Sep 30;22(19):10595. doi: 10.3390/ijms221910595 (PMC8508844; doi:10.3390/ijms221910595)
Supplement: Supplementary file 1 [file ijms-22-10595-s001.zip › ijms-1380781-supplementary.pdf]

Supplementary Materials

Engineering efficient self-assembled plasmonic nanostructures by configuring metallic nanoparticle's morphology

Vasanthan Devaraj [1,†], Jong-Min Lee [1,2,†] , Ye-ji Kim [3] , Hyuk Jeong [1] and Jin-Woo Oh [1,3,4,*]

[1]Bio-IT Fusion Technology Research Institute, Pusan National University, Busan, 46241, Republic of Korea; devarajvasanthan@gmail.com

[2]School of Nanoconvergence Technology, Hallym University, Chuncheon, 24252, Republic of Korea; jongminlee1984@gmail.com

[3]Department of Nano Fusion Technology and BK21 Plus Nano Convergence Division, Pusan National University, Busan, 46241, Republic of Korea; kkyeaj0608@gmail.com

[4]Department of Nanoenergy Engineering, Pusan National University, Busan, 46241, Republic of Korea

*Correspondence: ojw@pusan.ac.kr

[†]V.D. and J.-M.L contributed equally.

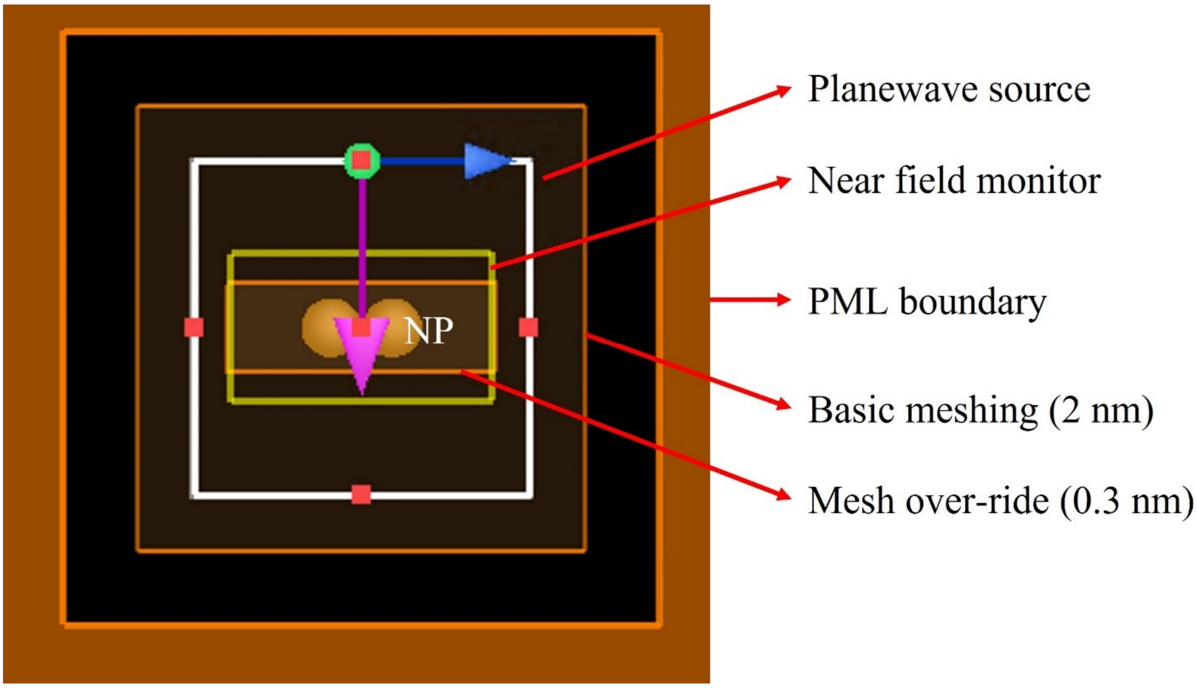

Figure S1. Modelling information which is used throughout our simulations.

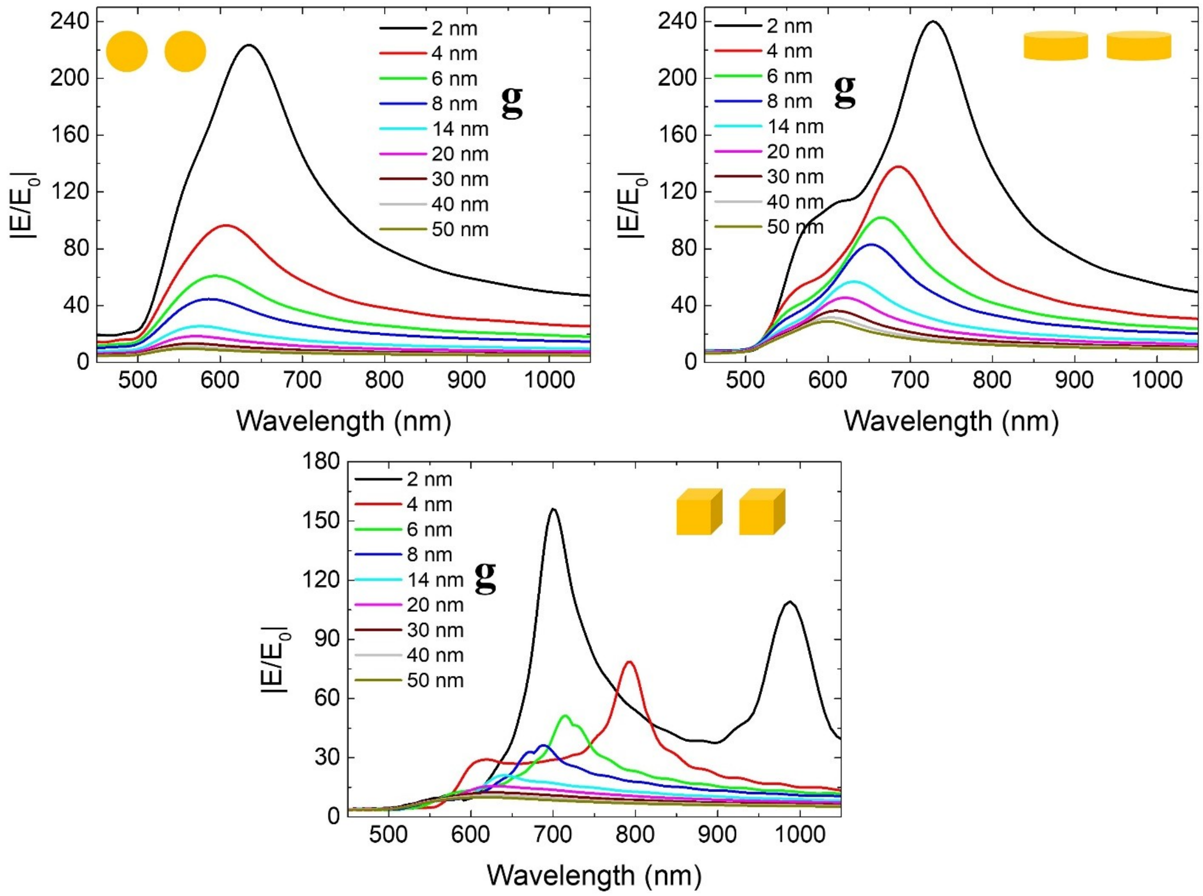

Figure S2. Simulated broadband near field spectra $|E/E_0|$ for dimer nanostructures with NP shapes of a sphere (**a**), disk (**b**), and cube (**c**) as a function of gap size.

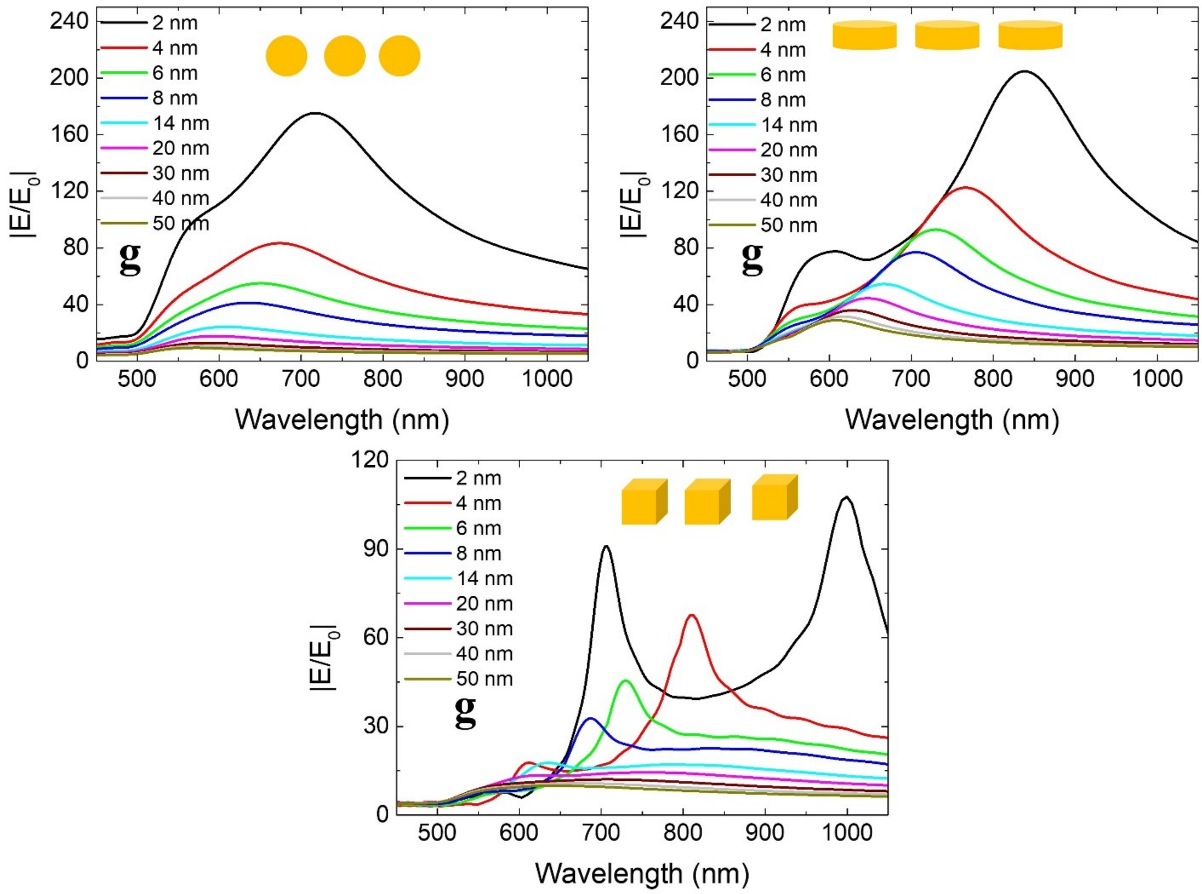

Figure S3. Simulated broadband near field spectra $|E/E_0|$ for trimer nanostructures with NP shapes of a sphere (**a**), disk (**b**), and cube (**c**) as a function of gap size.

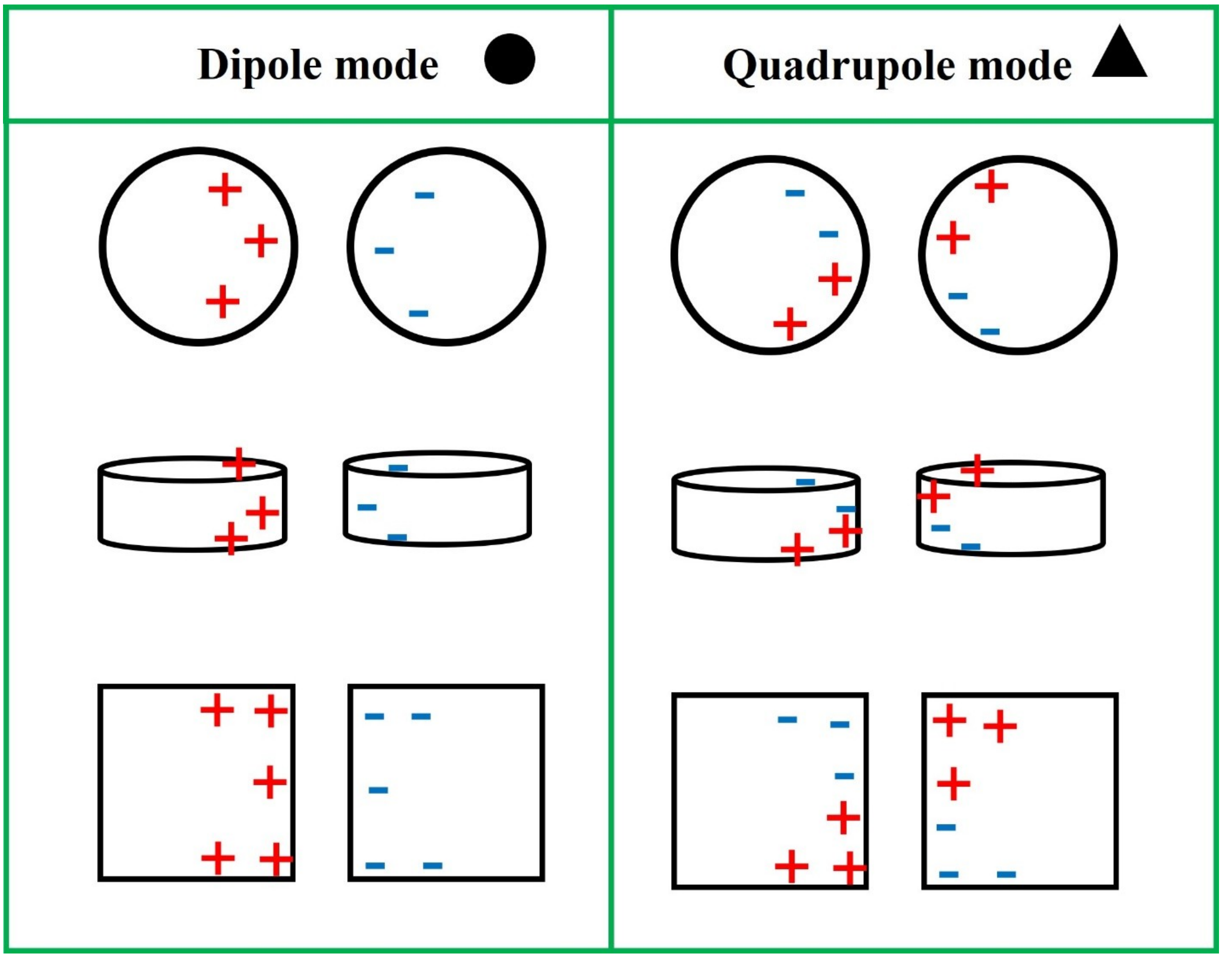

Figure S4. Schematic description of surface charge distributions reveals dipolar or quadrupolar modes in a plasmonic cavity region.

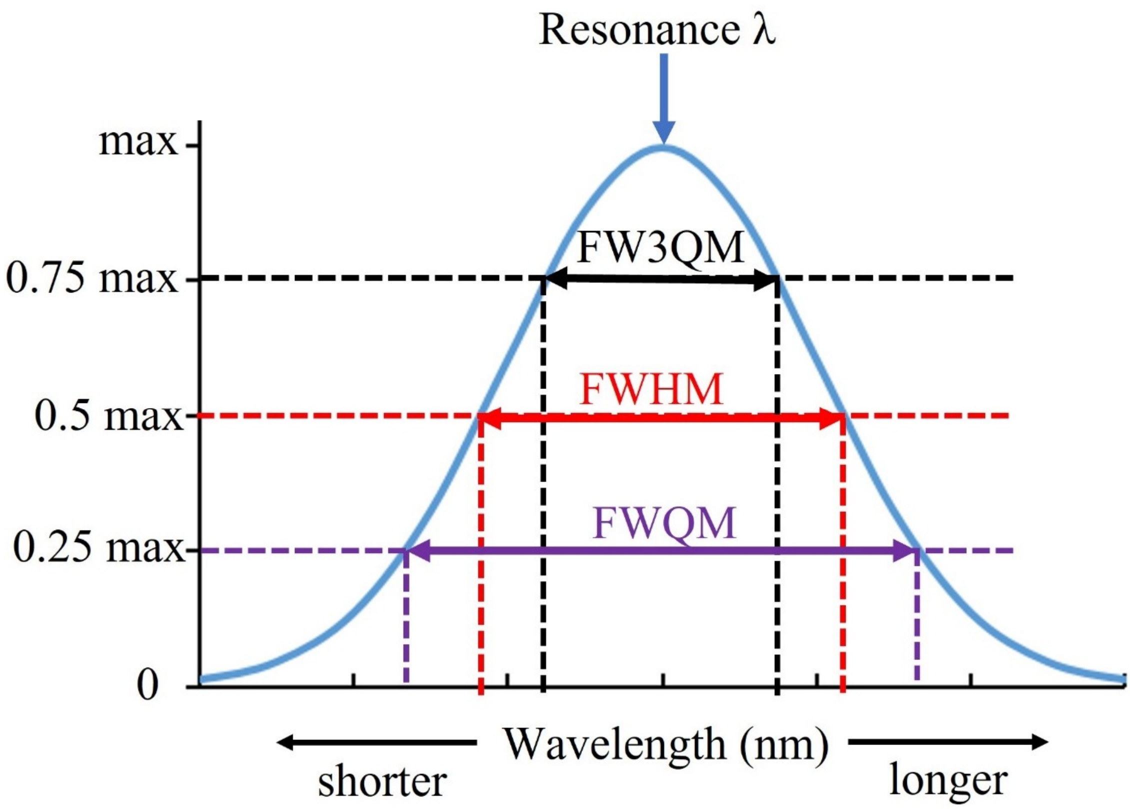

Figure S5. Data covering the FW3QM region in evaluating the broadband spectral performance of plasmonic nanostructures.

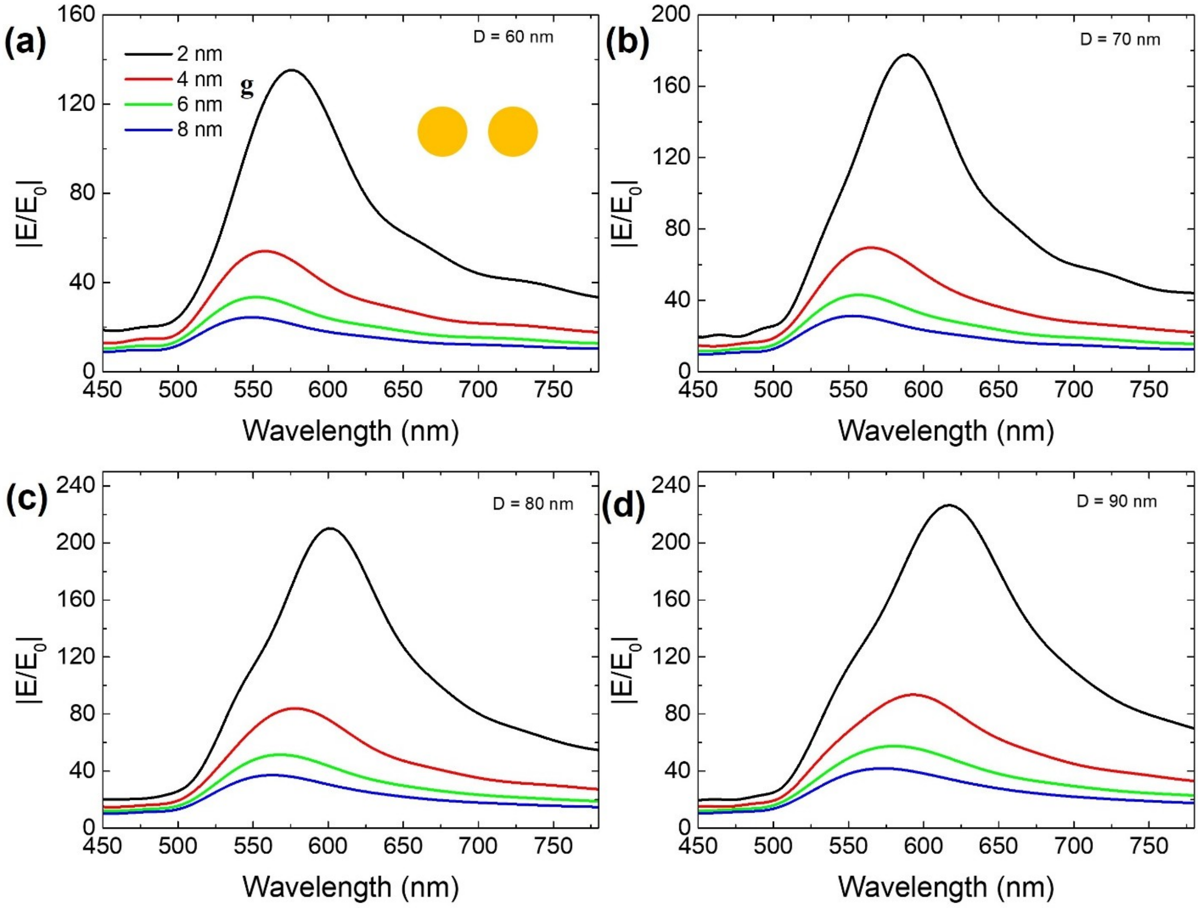

Figure S6. Simulated broadband gap-size dependent near field spectra $|E/E_0|$ for sphere-based dimer nanostructures with different NP diameter: 60 nm (**a**), 70 nm (**d**), 80 nm (**c**) and 90 nm (**d**).

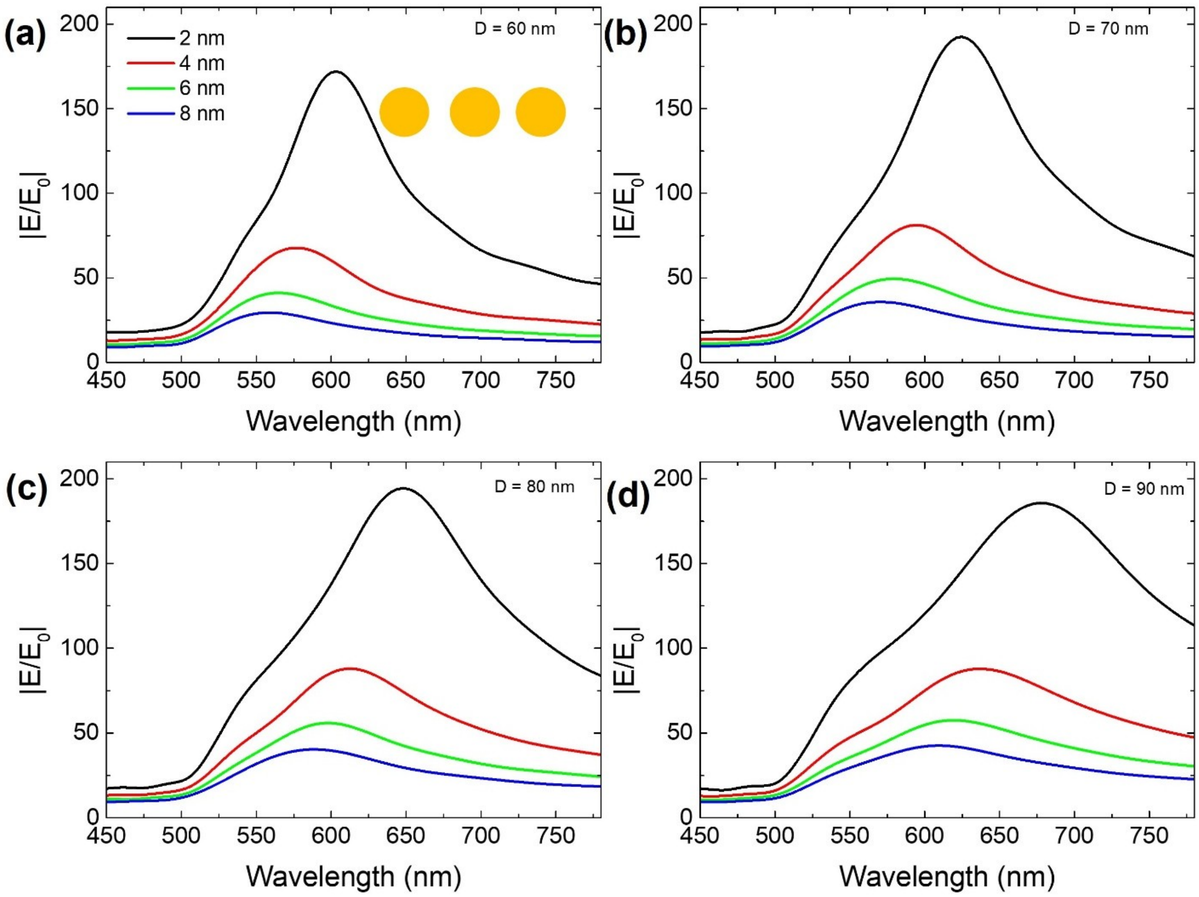

Figure S7. Simulated broadband gap-size dependent near field spectra $|E/E_0|$ for sphere-based trimer nanostructures with different NP diameter: 60 nm (**a**), 70 nm (**d**), 80 nm (**c**) and 90 nm (**d**).

Gap size (nm)	Max $\|E/E_0\|$ at Resonance λ	75% of max $\|E/E_0\|$	75% of max $\|E/E_0\|$ at shorter λ (nm)	75% of max $\|E/E_0\|$ at longer λ (nm)	FW3QM (nm)
Sphere dimer					
2	223	167.25	587	684	97
4	97	72.75	559	662	103
6	61	45.75	548	656	108
8	45	33.75	546	651	105
Disk dimer					
2	240	180	685	771	86
4	138	103.5	645	731	86
6	102	76.5	626	708	82
8	83	62.25	614	696	82
Cube dimer					
2	109	81.75	963	1014	51
4	79	59.25	775	810	35
6	52	39	699	741	42
8	36	27	658	716	58

Table S1. FW3QM calculation information for dimer nanostructures for different gap sizes.

Gap size (nm)	Max $\|E/E_0\|$ at Resonance λ	75% of max $\|E/E_0\|$	75% of max $\|E/E_0\|$ at shorter λ (nm)	75% of max $\|E/E_0\|$ at longer λ (nm)	FW3QM (nm)
Sphere trimer					
2	175	131.25	637	806	169
4	84	63	596	769	173
6	55	41.25	577	752	175
8	42	31.5	568	736	168
Disk trimer					
2	205	153.75	775	909	134
4	123	92.25	708	836	128
6	93	69.75	674	797	123
8	77	57.75	653	772	119
Cube trimer					
2	108	81	970	1031	61
4	68	51	788	832	44
6	46	34.5	711	756	45
8	33	24.75	667	719	52

Table S2. FW3QM calculation information for trimer nanostructures for different gap sizes.
